# Elevated urine oxalate and renal calculi in a classic galactosemia patient on soy‐based formula

**DOI:** 10.1002/jmd2.12056

**Published:** 2019-06-21

**Authors:** Julia A. Sabatino, Danielle Starin, Shamir Tuchman, Carlos Ferreira, Debra S. Regier

**Affiliations:** ^1^ Genetics and Metabolism Rare Disease Institute, Children's National Medical Center Washington District of Columbia; ^2^ Department of Nephrology Children's National Medical Center Washington District of Columbia; ^3^ National Human Genome Research Institute, National Institutes of Health Bethesda Maryland

**Keywords:** galactose‐1‐phosphate uridylyltransferase, galactosemia, nephrocalcinosis, oxalate, soy, stone

## Abstract

Classic galactosemia results from a deficiency in the galactose‐1‐phosphate uridylyltransferase (GALT) enzyme, which is essential for galactose metabolism. Treatment focuses on lactose restriction and is achieved with a soy‐based diet. Previously, renal calculi have not been documented in galactosemia patients. We present a patient with galactosemia nutritionally dependent on soy‐based formula via G‐tube, who developed renal calculi. Analysis of urinary stone risk factors revealed elevated urine oxalate levels and stone analysis confirmed calcium oxalate composition. Initiation of lactose‐ and soy‐free elemental formula returned urine oxalate level to normal. Given the presence of a metabolic pathway for the conversion of galactose to oxalate, and the high content of oxalate in soy‐based formula, there is the potential for elevated urine oxalate and a resulting risk of urinary calculi formation in patients with classic galactosemia. This potential can be effectively managed with a lactose and soy‐free elemental diet. This report describes the clinical course and novel findings of calcium oxalate urinary calculi in a classic galactosemia patient dependent upon soy‐based formula, with a discussion regarding the multiple factors leading to increased stone formation in this patient.

## 1 INTRODUCTION

Galactosemia disorders encompass three known inborn errors of galactose metabolism derived from deficiency in the enzymes galactokinase (GALK1), galactose‐1‐phosphate uridylyltransferase (GALT), and UDP‐galactose 4‐epimerase (GALE).^1^, ^2^ Classic galactosemia is an autosomal recessive disorder affecting 1 in 40 000‐60 000 births, caused by complete or partial deficiency of the GALT enzyme. Deficiency in galactose metabolism poses an early and life threatening challenge to a newborn since galactose and glucose are the two component sugars of lactose, the primary carbohydrate in both dairy and breast milk. Manifestations of life‐threatening symptoms appear within days of beginning feeding with breast or formula milk. Early signs include feeding difficulties, failure to thrive, vomiting, lethargy, bleeding abnormalities, and jaundice (Reviewed in^1^, ^3^). Life‐threatening sepsis and meningitis, often caused by *Escherichia coli*, and cataracts can develop.

The GALT enzyme is responsible for converting galactose‐1‐phosphate to UDP‐galactose in the galactose metabolic pathway.^2^ Deficiency results in elevated galactose, galactose‐1‐phosphate, galactitol, and galactonate levels. Newborn screening can detect galactosemia by measurement of GALT enzyme activity and total galactose level. Confirmatory testing for classic galactosemia includes levels of erythrocyte galactose‐1‐phosphate and genetic testing for mutations in the *GALT* locus on chromosome 9.^3^

Treatment of classic galactosemia is urgent in the first few days of life and includes immediate lactose restriction by switching to soy‐based formula. Lactose restriction results in rapid clinical improvement.^1^, ^3^ However, galactose is obtained through both dietary sources and by endogenous synthesis, therefore even in patients with no lactose intake from the diet, levels of erythrocyte galactose‐1‐phosphate remain elevated. Long‐term outcomes in patients with treated galactosemia include mild growth delay, decreased bone density, delayed speech development, verbal dyspraxia, cerebellar ataxia, and dystonia (Reviewed in ref.^1^). These complications are considered diet independent and can appear in patients with well‐controlled erythrocyte galactose‐1‐phosphate level.

While not previously described in the galactosemia population, nonambulatory patients are at increased risk for kidney stones. The underlying mechanism appears to be the decreased mineralization of bone, leading to increased urinary excretion of calcium. In addition, low volume intake can lead to increased stone formation, which can be seen in nonambulatory individuals on G‐tube feeding plans.

Here, we present a patient with classic galactosemia and a history of *E. coli* meningitis as a neonate that lead to severe, global delays. Following dependence on soy‐based formula via gastric tube (G‐tube), he developed calcium oxalate urinary calculi. Previously galactosemia and renal calculi have not been associated, however, we will demonstrate a clinical course and metabolic pathway that intertwines these conditions.

## 2 CLINICAL CASE

Our patient is a 14‐year‐old male with classic galactosemia with significant neurologic injury caused by neonatal septicemia with *E. coli*. He has severe mobility limitations, leading to wheelchair confinement, and oral feeding intolerance, leading to dependency on gastric‐tube nutrition. He was placed on a soy‐based formula. Additionally, he is status postplacement of two ventriculoperitoneal shunts for hydrocephalus with a history of seizures, kyphoscoliosis, and optic atrophy without cataracts.

Following a trauma event, he returned to care at a center with metabolic specialists at age 9 years. GALT enzyme activity was tested to determine optimal dietary options and was consistent with classic galactosemia (Gal‐1‐P Uridyltransferase <2.0 μmol/hr/mmol Hb, reference range > 18.5 μmol/hr/mmol Hb). Erythrocyte galactose‐1‐phospate was in the treatment range. Soy‐based formula was continued to meet his nutritional needs at this time.

The patient represented at age 12 with repeated episodes of urinary retention treated by catheterization. Repeated renal/bladder ultrasounds and a noncontrast abdominal CT scan over the subsequent 2 years indicated urinary calculi within the kidneys and bladder wall. The largest imaged stone was 1 cm in diameter in the bladder. The patient presented with recurring symptoms of stone passage and was treated for repeated urinary tract infections/colonization with *Enterococcus faecalis*. Throughout this time, renal/bladder ultrasounds continued to demonstrate renal and bladder calculi, the patient's erythrocyte galactose‐1‐phosphate was in the treatment range (3.1 mg/100 mL RBC, reference range for treated galactosemia <15), and he remained on soy‐based formula.

Given persistent urinary calculi formation, additional testing was performed and showed minimal elevation of 25‐hydroxy vitamin D3 (56 ng/mL, normal range 10‐44 ng/mL), and spot random urine oxalate to creatinine ratio (99 mg/g creatinine [Cr], reference range < 75 mg/g Cr). At this time, supplementation with cholecalciferol was discontinued. On follow‐up, the patient continued to demonstrate persisting urinary calculi on ultrasound and an elevated urine oxalate to creatinine ratio (259 mg/g Cr, reference range < 75 mg/g Cr).

Soybeans contain relatively high levels of oxalic acid.^4^, ^5^, ^6^, ^7^ Oxalate content of foods is not reported by food manufacturers and oxalate levels are also not standard in the food nutrient analysis by the USDA for the food composition database. For this reason, it is difficult to determine the exact amount of oxalate in soy‐based foods and formulas he was receiving. Two studies measured oxalate content in soymilk and showed a range from 0.02 to 1.4 mg/g protein.^5^ Extrapolating this range to the pediatric soy drink (since information not available from the manufacturer) indicated he was receiving 24‐1680 g of oxalate per day. Cow's milk has been estimated by an online resource as being low to very low with a range of 0.004‐0.02 g/g protein.^8^ Thus, soy milk has up to 3500‐fold more oxalate per gram than cow's milk. Based on the patient's unique dependence on purely soy‐based formula, a change to a lactose‐ and soy‐free elemental formula was performed as a trial. Following this change, urine oxalate levels quickly fell within normal limits (49 mg/g Cr). Additionally, retrieval and stone analysis demonstrated calculi composition to be 50% calcium oxalate and 50% carbonate apatite.

## 3 DISCUSSION

**Figure 1:**
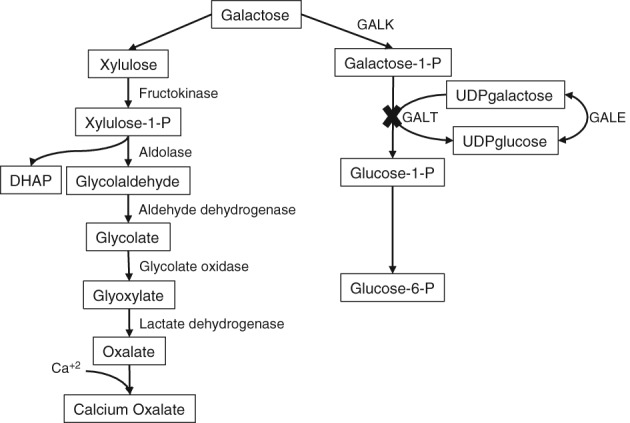
Proposed mechanism for the formation of oxalate as an alternative pathway of galactose metabolism in Classic Galactosemia. Blocked metabolism of galactose is shown by the deficiency in galactose‐1‐phosphate uridyltransferase (GALT). Galactose metabolism can proceed by conversion to xylulose by the enzyme galactose dehyrogenase. Xylulose is converted to xylulose‐1‐phosphate by the enzyme fructokinase and then to glycolaldehyde and dihydroxyacetone phosphate (DHAP) by an aldolase. Glycolaldehyde is first converted to glycolate by aldehyde dehydrogenase and then to glyoxylate by glycolate oxidase before final conversion to oxalate by lactate dehydrogenase. The addition of calcium results in the formation of calcium oxalate

Failure of the GALT enzyme in galactosemia leads to accumulation of galactose in the body. This build up can be managed effectively with restriction of lactose, although levels will not be as low as in the general population (Reviewed in^1^). In cases when the conversion of galactose to glucose is impaired, as in galactosemia, excess galactose has been shown to favor an alternative direct oxidative pathway.^9^ The presence of this alternative oxidative pathway in galactosemic patients was proven by intravenous injection of a radioactive galactose tracer, followed by measurement of radioactively labeled CO_2_ in expired air.^10^ This pathway purportedly leads to the formation of D‐xylulose,^11^ while the latter is eventually converted to oxalate^10^, ^12^ (Figure 1). Activity of the first committed enzyme in this proposed pathway, that is, galactose dehydrogenase, has been found in mammalian liver‐including human liver‐with 90% of the activity present in the soluble cellular fraction, and 10% in the microsomal fraction.^11^ The second branch of the pathway, from D‐xylulose to oxalate, was found in human liver based on radioactive tracer studies and enzyme assays; these studies suggested that the major regulation in the conversion of carbohydrates to oxalate occurs at the level of the enzymes participating in glycolate and glyoxylate metabolism.^10^ Although the final products of these two halves of the oxidative pathway have been confirmed, the intermediate steps remain hypothetical, especially until further delineation at the molecular level can be established.

Additionally, this patient had an exclusively soy‐based diet. Since soy foods contain relatively high levels of oxalate;^5^, ^6^, ^7^ thus, he had both increased intake and increased proposed production of oxalate. Taylor and Curhan [11] showed oxalate intakes were 214 mg/d in men, 185 mg/d in older women, and 183 mg/d in younger women. Thus, this patient had up to 8 times the average intake of oxalate for the typical male.

Further, supplementation with calcium and vitamin D are recommended in classic galactosemia patients to prevent bone demineralization.^1^ This may lead to increased urinary calcium excretion due to impaired bone mineral accrual, especially in patients that are not ambulatory. Together, increased urine oxalate and calcium excretion heightens the potential for calcium oxalate urinary calculi formation.

This case may represent a discrete subset of patients with classic galactosemia, who are solely dependent on soy‐based formula. In addition, he had elevated risks for renal stones due to decreased mobility and lack of weight‐bearing exercise. Thus, this sub‐population of patients with galactosemia are at an even higher than expected risk for stone formation. We demonstrate that a simple intervention of a dietary change to lactose‐ and soy‐free elemental formula can effectively reduce urine oxalate levels.

Although there are many long‐term sequelae associated with treated galactosemia, urinary calculi have not previously been documented. This case demonstrates a proposed connection between galactosemia and urinary calculi formation and highlights the need to consider urinary calculi and urine oxalate levels in the galactosemia patient population, and particularly in those who are solely dependent on soy‐based formula and/or nonambulatory.

## 4 CONCLUSIONS

Classic galactosemia is present in 1 in 40 000‐60 000 newborns. Immediate lactose restriction is extremely important in preventing life threatening multiorgan damage in these patients. Patients with classic galactosemia are known to metabolize excess galactose via an alternative oxidative pathway that gives rise to D‐xylulose, which in turn is converted to oxalate. When utilizing a soy‐based formula to treat patients with classic galactosemia, elevated urine oxalate levels may indicate the potential for urinary calculi formation and should be monitored. Modification of the diet to lactose‐ and soy‐free elemental formula can significantly reduce urine oxalate levels and should be considered for galactosemia patients with elevated kidney stone risk based on other risk factors.

## AUTHOR CONTRIBUTIONS

Julia A Sabatino provided clinical care and drafted the manuscript. Danielle Starin provided clinical care and edited the manuscript. Shamir Tuchman provided clinical care and edited the manuscript. Carlos Ferreira provided extensive discussion and laboratory knowledge and edited the manuscript. Debra S Regier provided clinical care and wrote and edited sections of the manuscript.

## CONFLICT OF INTEREST

The authors declare no potential conflict of interest.

## A PATIENT CONSENT STATEMENT

The family of the patient described provided informed consent for publication of this case.
